# Proposal of a Mediterranean Diet Serving Score

**DOI:** 10.1371/journal.pone.0128594

**Published:** 2015-06-02

**Authors:** Celia Monteagudo, Miguel Mariscal-Arcas, Ana Rivas, María Luisa Lorenzo-Tovar, Josep A. Tur, Fátima Olea-Serrano

**Affiliations:** 1 Research Group Nutrition, Diet and Risk Assessment (AGR-255), Department of Nutrition and Food Science, University of Granada, Campus of Cartuja s/n, 18071, Granada, Spain; 2 Department of Food Technology, Nutrition and Food Science, University of Murcia, Campus de Lorca, 30800, Lorca, Spain; 3 Research Group on Community Nutrition and Oxidative Stress, University of Balearic Islands & CIBEROBN (Physiopathology of Obesity and Nutrition), E-07122, Palma de Mallorca, Spain; National Institute of Agronomic Research, FRANCE

## Abstract

**Background and Aims:**

Numerous studies have demonstrated a relationship between Mediterranean Diet (MD) adherence and the prevention of cardiovascular diseases, cancer, and diabetes, etc. The study aim was to validate a novel instrument to measure MD adherence based on the consumption of food servings and food groups, and apply it in a female population from southern Spain and determining influential factors.

**Methods and Results:**

The study included 1,155 women aged 12-83 yrs, classified as adolescents, adults, and over-60-yr-olds. All completed a validated semi-quantitative food frequency questionnaire (FFQ). The Mediterranean Dietary Serving Score (MDSS) is based on the latest update of the Mediterranean Diet Pyramid, using the recommended consumption frequency of foods and food groups; the MDSS ranges from 0 to 24. The discriminative power or correct subject classification capacity of the MDSS was analyzed with the *Receiver Operating Characteristic* (ROC) curve, using the MDS as reference method. Predictive factors for higher MDSS adherence were determined with a logistic regression model, adjusting for age. According to ROC curve analysis, MDSS evidenced a significant discriminative capacity between adherents and non-adherents to the MD pattern (optimal cutoff point=13.50; sensitivity=74%; specificity=48%). The mean MDSS was 12.45 (2.69) and was significantly higher with older age (p<0.001). Logistic regression analysis showed highest MD adherence by over 60-year-olds with low BMI and no habit of eating between meals.

**Conclusions:**

The MDSS is an updated, easy, valid, and accurate instrument to assess MD adherence based on the consumption of foods and food groups per meal, day, and week. It may be useful in future nutritional education programs to prevent the early onset of chronic non-transmittable diseases in younger populations.

## Introduction

The Mediterranean Diet (MD) is a dietary pattern established in countries of the Mediterranean Basin in the mid-1950s in a situation of severe economic difficulties and resource limitations due to the effects of World War II. These conditions, alongside a low level of technology, favored a physically active and a frugal lifestyle, with a predominance of vegetable products and scarcity of animal-derived products in the diet [1]. Many of these life habits have been preserved and are part of our dietary traditions, being protected by the UNESCO as an Intangible World Cultural Heritage [2].

Current studies show that Mediterranean populations generally meet around 50% of MD recommendations [3–8]. Numerous indexes or scores have been applied to measure adherence to this dietary pattern. The first and most widely used is the *Mediterranean Dietary Score* (MDS), proposed by Trichopoulou et al. [9,10], which assesses the compliance with this dietary pattern in adults, including the elderly, assigning 1 point when the intake of protective foods in the MD is above the median or when the intake of non-protective foods is below the median, and 0 points in the opposite situations. Other indexes based on the MDS have been proposed that are adapted to specific situations and/or use a different calculation approach. Examples include: KIDMED [11], which assesses adherence to the MD among children and adolescents; the MDS-p designed for pregnant women, which considers micronutrients especially relevant during pregnancy, such as iron, calcium, and folic acid [5]; the Breakfast Quality Index (BQI), which assesses the quality of this meal in the context of the MD [12]; the MDP, which calculates the percentage adherence to the MD pattern using the Z value [13]; and, most recently, the relative Mediterranean Diet Score (rMDS), based on the nutrient density model with intake divided into tertiles [14].

Application of these indexes has revealed changes in dietary habits, based on differences among the three or four generations coexisting in given populations. Thus, a greater adherence to this dietary pattern has been shown by elderly people than by younger age groups [14,15]. The former preserve conventional cooking and eating habits, preparing recipes based on legumes, vegetables, potatoes, olive oil, and cereals, etc., eating bread with meals and fruit as dessert. Moreover, meals are not only regarded as necessary for nutritional intake but also as an important family and social event [16]. In contrast, the eating habits of younger populations (children and adolescents) are closer to Western dietary patterns, with a higher consumption of fats and proteins than recommended, to the detriment of carbohydrate intake, and a deficient intake of vitamins and minerals [15]. Along with a less physically active lifestyle than in the past, these changes have been related to the early onset of non-transmittable chronic diseases such as obesity, type II diabetes, hyperlipidemia, cardiovascular diseases, metabolic syndrome, and some types of cancer [17,18].

The new MD pyramid has been adapted to specific national settings (e.g., in relation to portion sizes) and to variations in the dietary pattern among different Mediterranean regions and cultures. In view of this recent update, the aim of this study was to propose and validate a new instrument to measure MD adherence based on the consumption of food servings and food groups, and apply it in a female population from southern Spain and determining influential factors.

## Material and Methods

### Study Population

Females aged between 12 and 83 years (n = 1155) were recruited between 2005 and 2009 from participants in a research project in Southern Spain in which a very small minority of volunteers were males, explaining the decision to enroll only females. They had all participated in different research studies under the auspices of the Health Department of Granada City Council and University of Granada. The study population was divided into three generational groups: Group 1, adolescents aged between 12 and 19 yrs (n = 610); Group 2: adults between 20 and 60 yrs (n = 313); and Group 3: over 60-year-olds (n = 232).

The study was conducted according to the guidelines laid down in the Declaration of Helsinki, and all procedures involving human subjects were approved by the ethics committee of the University of Granada. Written informed consent was obtained from all participants as well as from the parents or guardians of minors.

### Questionnaire

After obtaining informed consent to participate in the study, trained researchers used a self-administered questionnaire to gather data from each participant on the following variables: age, weight and height using a model 872 Seca digital floor scale and a model 214 Seca portable stadiometer (Seca Medical Scales and Measuring Systems, Birmingham, UK), socioeconomic variables (educational level, profession), and dietary habit variables, including: number of meals/day, place of meal, special diet (e.g., weight-reducing or therapeutic diet) during the previous year, habit of snacking between meals, consumption of “diet” products) (Table 1). All participants completed a semi-quantitative food frequency questionnaire (FFQ) previously validated by our research group [8,19,20]. It includes foods commonly consumed in the Mediterranean area, recording the frequency of consumption of the food items over the previous 12 months as: never, less than once/month; once/month; 2–3 times/month; 1–2 times/week; 3–4 times/week; 5–6 times/week; once/day; 2–3 times/day; 4–5 times/day. The amounts of food consumed were expressed in g, mL, domestic measures (e.g., slice, tablespoon, cup), or standard portions [21]. The questionnaire included 129 items classified into 11 food groups (cereals, vegetables, fruit, eggs, meat, fish, fats, prepared foods, sauces, alcohol-free drinks, and alcoholic drinks) and required a mean time of 30 min to complete. The daily intake of each nutrient was calculated by multiplying the amount reported in the questionnaire by the corresponding value in the food composition table [21,22]. Foods were converted into nutrients using a computer program (DIAL 1.0 Programa para Evaluación de Dietas y cálculos de Alimentación, 2008 ALCE Ingeniería, Las Rozas, Madrid, Spain).

**Table 1:** *Mediterranean Diet Serving Score* (MDSS).

	Recommendation*	Score
Fruit	1–2 servings/main meal**	3
Vegetables	≥ 2 servings/main meal**	3
Cereals^a^	1–2 servings/main meal**	3
Potatoes	≤ 3 servings/week	1
Olive Oil^b^	1 serving/main meal**	3
Nuts	1–2 servings/day	2
Dairy products^c^	2 servings/day	2
Legumes	≥ 2 servings/week	1
Eggs	2–4 servings/week	1
Fish	≥ 2 servings/week	1
White meat^d^	2 servings/week	1
Red meat^e^	< 2 servings/week	1
Sweets^f^	≤ 2 servings/week	1
Fermented beverages^g^	1–2 glass/day	1
**Total score**		**24**

* According with the new Mediterranean Diet Pyramid [16].

** Main meals: breakfast, lunch and dinner.

^a^ Bread, breakfast cereals, rice and pasta.

^b^ Olive oil used on salads or bread or for frying

^c^ Milk, yoghurt, cheese, ice-cream

^d^ Poultry

^e^ Pork, beef, or lamb

^f^ Sugar, candies, pastries, sweetened fruit juices, and soft drinks

^g^Wine and beer.

### Mediterranean Dietary Serving Score (MDSS)

The proposed Mediterranean Dietary Serving Score (MDSS) is based on the latest update of the Mediterranean Diet Pyramid [16], using the recommended consumption frequency of different foods and food groups. Individuals whose intake is within the number of recommended servings are awarded a score of 3, 2, or 1 points for recommendations expressed in times/meal, times/day, or times/week, respectively. This approach gives greater importance to foods that should be consumed in every meal (fruit, vegetables, olive oil, cereals), followed by those that should be consumed daily (dairy products and dried fruit and nuts), and finally, those that should be consumed weekly (potatoes, legumes, eggs, fish, white meat, red meat, sweets). In adults, 1 point is added for alcohol intake equivalent to 1 and 2 glasses of wine or beer (fermented drinks) for females and males, respectively. A score of 0 is given when the number of servings/meal, week, or day is higher or lower than the recommendation. Hence, the MDSS ranges between 0 and 24 points for adults/elderly, and between 0 and 23 for adolescents, because no level of fermented beverages consumption is considered positive in this age group (Table 1).

### Statistical analysis

A descriptive analysis was conducted to compute means with standard deviation (SD) for quantitative variables and frequencies (%) for qualitative variables. The Receiver Operating Characteristic (ROC) curve was constructed to analyze the discriminative power or correct subject classification capacity according to the MDSS. The reference method was the MDS [9,10], which ranges from 0 to 9; the cutoff point of 6 selected to differentiate MD adherents and non-adherents was that proposed by most authors for this purpose [23–26]. The sensitivity of the MDSS was calculated as the ratio of true positives to true positives plus false negatives; the specificity was calculated as the ratio of true negatives to true negatives plus false positives [27]. We also used the t-test for independent samples to compare the mean number of servings consumed between tertiles 1 and 3 and an ANOVA to compare the mean number of servings consumed among the three age groups. Finally, logistic regression analysis was used to determine the factors predicting the highest adherence to MDSS, adjusting for age. The highest adherence was defined as an MDSS above the cut-off point of the third tertile (MDSS≥16). P<0.05 was considered significant.

## Results

The general characteristics of the study population sample are shown in Table 2. The mean age was around 13 yrs for adolescents, 44 yrs for adults, and 70 yrs for elderly women. There were significant weight differences among the three age groups and the BMI of the elderly women group was the closest to the obesity cut-off point. Most elderly women had low educational level and were housewives. Almost 50% of the adolescents had 5 meals a day, while the most adult and elderly women had between 3 and 4 meals a day. Most of the sample ate at home; more frequently in adults and elderly women (>90%) than in adolescents (74.3%). Most of the study sample had not followed any special weight-loss or therapeutic diet over the past few years and did not have a habit of snacking between meals.

**Table 2:** Baseline characteristics of the study sample.

		12 to 19 yrs (n = 610)	20 to 60 yrs (n = 313)	>60 yrs (n = 232)
Age (years)	Mean (SD)	12.82 (0.84)	44.02 (10.46)	70.19 (5.99)
Weight (Kg)	Mean (SD)	50.36 (11.10)	65.74 (10.79)	70.90 (9.46)
Height (m)	Mean (SD)	1.58 (0.10)	1.61 (0.06)	1.57 (0.13)
BMI (Kg/m^2^)	Mean (SD)	19.88 (2.87)	22.38 (4.37)	28.50 (3.68)
Educational level (%)	Low	-	27.6	70.4
	Medium	-	39.0	15.7
	High	-	33.3	13.9
Profession (%)	Low qualification	-	70.6	87.7
	High qualification	-	29.5	12.3
Meals/day (%)	≤3 meals/day	12.9	40.9	30.1
	4 meals/day	37.6	33.7	38.9
	≥5 meals/day	49.4	25.5	31.0
Habit of snacking between meals (%)	Yes	40.3	38.5	20.5
	No	59.7	61.5	79.5
Place of meal (%)	At home	74.3	90.4	95.4
	Out home	25.7	9.6	4.6
Special diet in the last year (%)	Weight-loss	8.3	14.3	14.9
	Therapeutic	1.2	7.8	10.5
	No	90.5	77.9	74.6
Diet food intake (%)	Daily	10.3	15.4	13.2
	Sometimes	47.5	51.1	20.7
	Never	43.2	33.4	66.1

Therapeutic diet: cholesterol, diabetes, allergies/food intolerance.

High-qualification occupation: managers/directors, professionals, scientists, office support staff; medium-low qualification occupation: service sector (catering, sales), craftspeople, construction, unskilled workers, homemakers.

**Fig 1:**
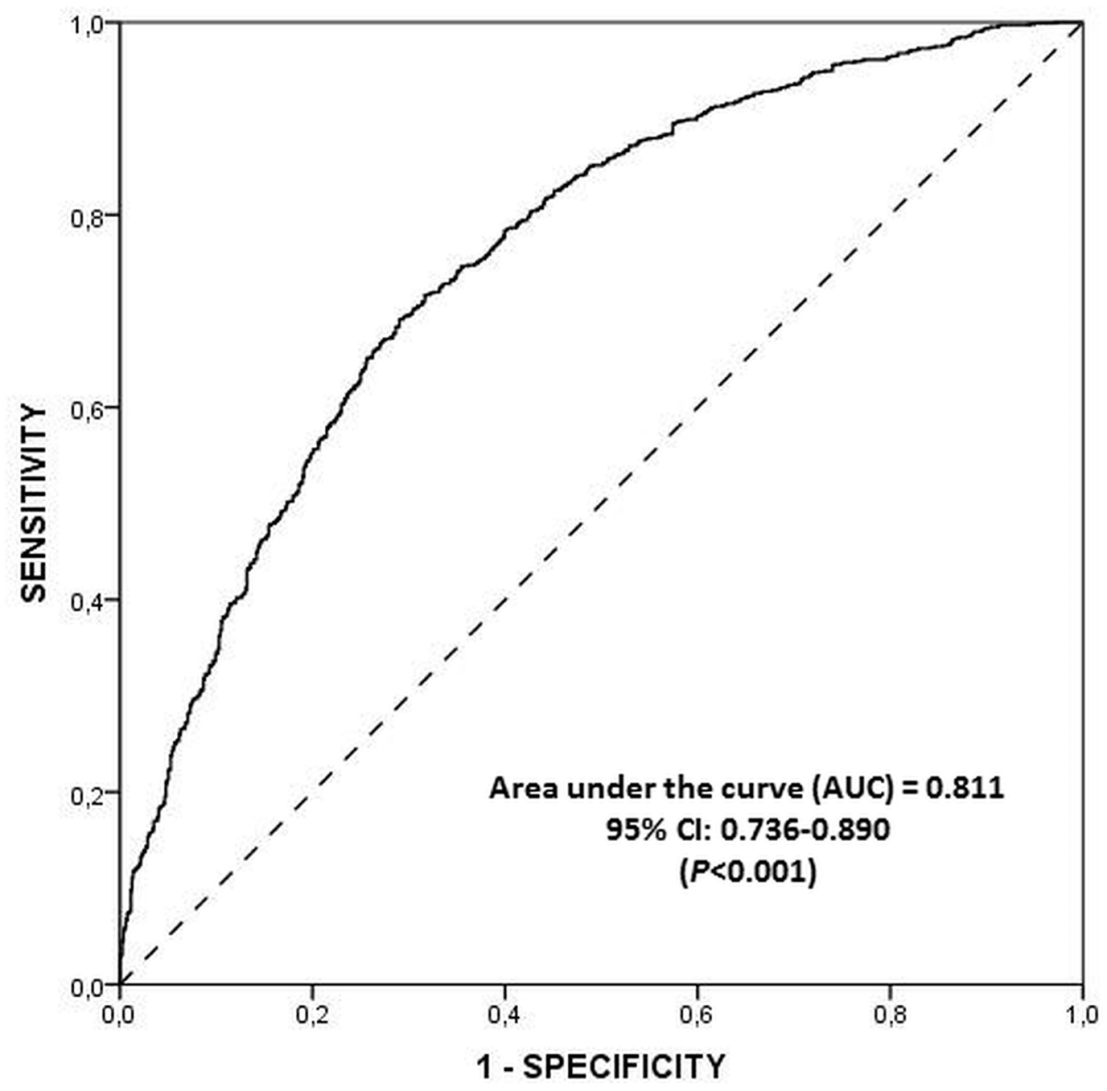
Discriminative power of the MDSS (ROC curve) using the MDS as reference method. The cutoff point for the reference method (MDS) was an MDS of 6, in agreement with other authors who considered that the upper tertile represents greater adherence to this dietary pattern [23–26].

Fig 1 shows the discriminative power of the MDSS, using the MDS as reference method. The MDSS demonstrated a discrimination capacity of 81% (Area under the ROC curve [AUC] = 0.811; 95% CI: 0.736–0.890). The MDSS that best discriminated between MD adherents and non-adherents (optimal cut-off point) was 15.50, with no statistically significant differences by age group (p = 0.230). The sensitivity for this cut-off value was 74% (95% CI: 72–75%) and the sensitivity was 48% (95% CI: 47–50%).

Mean MDSS and MDS values were 12.45 (2.69) and 4.58 (1.44) for the total population, and both values significantly increased with higher age (p<0.001) (Data not shown). Table 3 shows the population distribution according to MDSS and MDS cut-off points. Small differences (<10%) were found between the MDSS and MDS in the percentage of individuals classified within recommendations for fruit, cereals, legumes, and fish intake, which was higher in all cases with the MDSS. Larger differences were found for the vegetable and dairy product food groups, with 11.4% of individuals within recommendations for vegetable intake and 36% for dairy products according to the MDSS, whereas >50% were within recommendations for both food groups according to the MDS.

**Table 3:** Population distribution with respect to the cut-off points for intake within or outside recommendations according to the MDS and MDSS.

		*Within Recommendation* *	*Outside Recommendation* **	
			Low	Above
Fruit	MDS	71.0	29.0	-
	MDSS	78.9	21.1	0.0
Nuts	MDS	-	-	-
	MDSS	54.5	35.4	10.1
Vegetables	MDS	52.4	47.6	-
	MDSS	11.4	88.6	-
Cereals	MDS	46.6	53.4	-
	MDSS	50.1	49.9	0.0
Potatoes	MDS	-	-	-
	MDSS	77.3	-	22.7
Olive Oil	MDS	-	-	-
	MDSS	87.3	9.0	3.7
MUFA/SFA	MDS	53.5	46.5	-
	MDSS	-	-	-
Dairy products	MDS	52.5		47.5
	MDSS	36.0	11.5	52.5
Legumes	MDS	52.8	47.2	-
	MDSS	56.3	43.7	-
Eggs	MDS	-	-	-
	MDSS	65.7	20.0	14.3
Fish	MDS	69.1	30.9	-
	MDSS	73.4	26.6	-
Meat	MDS	40.9	-	59.1
	MDSS	-	-	-
White meat	MDS	-	-	-
	MDSS	24.8	18.3	56.9
Red meat	MDS	-	-	-
	MDSS	32.0	-	68.0
Sweets	MDS	-	-	-
	MDSS	63.4	-	36.6
Fermented beverages	MDS	18.8	81.2	-
	MDSS	18.8	81.2	1.2

* *Within recommendations* for MDS: intake > median for vegetables, legumes, fruits and nuts, cereals, MUFA/SFA, and fish; intake < median for meat, poultry, and dairy products; and fermented beverages intake between 5–25 g/day [10]. *Within recommendations* for MDSS: according to the food frequency recommended in the new Mediterranean Diet Pyramid [16].

** *Outside recommendations* for MDS: all other intakes. *Outside recommendation* for MDSS: below or above the food frequency recommended in the new Mediterranean Diet Pyramid [16].

**Fig 2:**
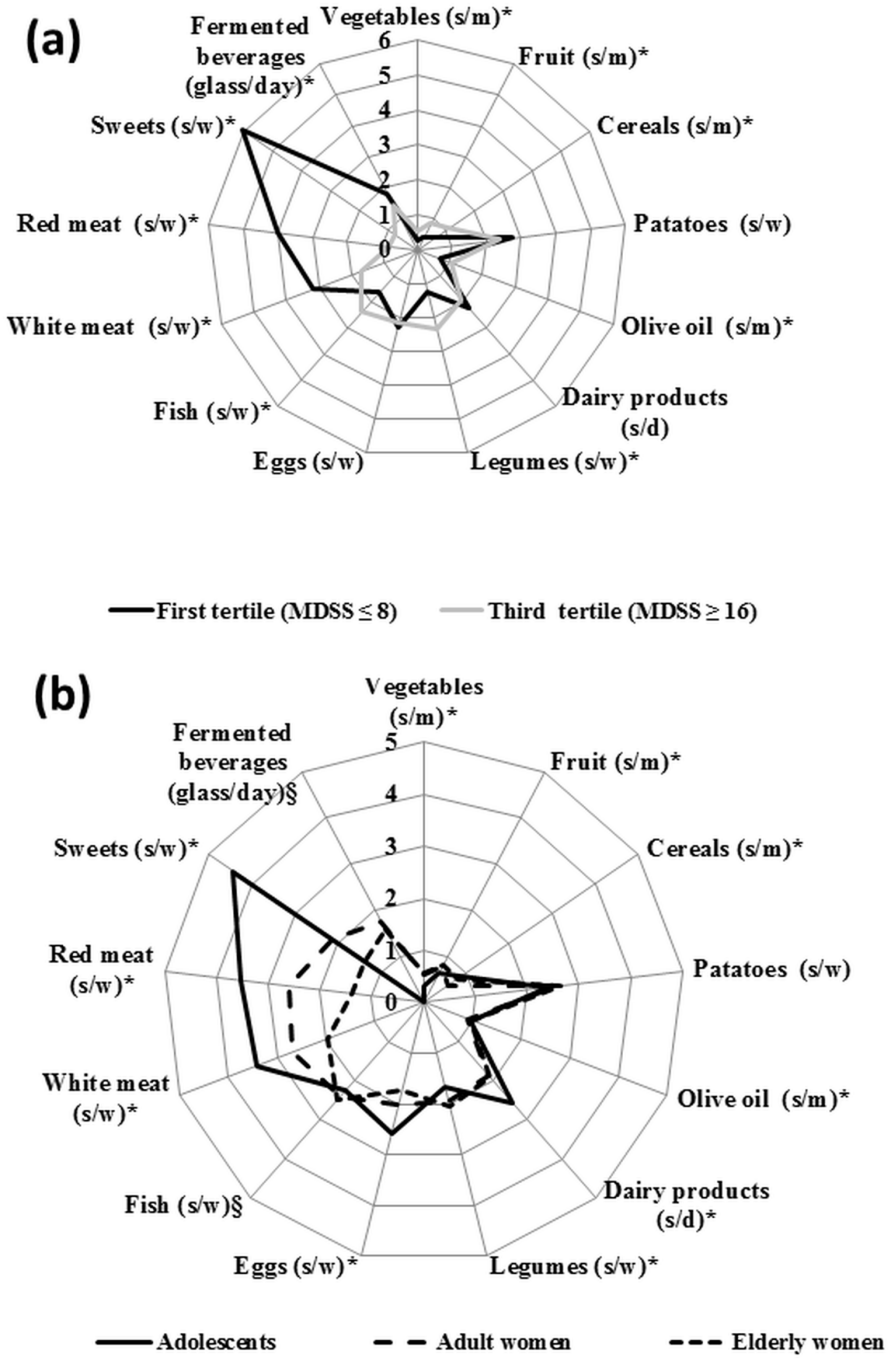
**(a)** Number of servings for each food group per meal, day, or week, according to MDSS tertiles. Differences by Student T-test: *p<0.05. s/m = serving/meal; s/d = serving/day; s/w = serving/week. **(b)** Number of servings for each food group per meal, day, or week for adolescent, adult, and elderly women. Differences by ANOVA: *p<0.001; ^§^p<0.05. s/m = serving/meal; s/d = serving/day; s/w = serving/week.

According to the first and third tertiles of the MDSS, the number of potato, dairy product, and egg servings was similar between these tertiles (p<0.05). However, there were significant differences for the other food groups. Thus, the intake of vegetables, fruit, olive oil, legumes, and fish was significantly higher in the third tertile, whereas the intake of meat (white and red), sweets, and fermented beverages was significantly higher in the first tertile. The number of servings consumed of each study food or food group significantly differed among age groups, except in the case of potatoes (p = 0.192). The number of fish and fermented beverage servings consumed also significantly differed among the three age groups, although with a less stringent significance level (p = 0.048 and p = 0.010, respectively) (Fig 2).

MDSS predictive factors were age, BMI, and habit of eating between meals. The highest adherence to the MD was shown by over-60-yr-old women with the lowest BMI and without the habit of snacking between meals (Table 4).

**Table 4:** Predictive factors for the highest adherence to MDSS (third tertile *vs*. other tertiles); logistic regression analysis, adjusting for age.

		Age-adjusted OR**	95% CI
Age group			
	Adolescents	Ref.	
	Adult women	2.39*	1.09–5.24
	Older women	7.68*	3.66–16.13
BMI***			
		0.91*	0.85–0.90
Educational level****			
	Low	Ref.	
	Medium	1.05	0.43–2.56
	High	1.07	0.54–2.10
Profession*****			
	High qualification	Ref.	
	Low qualification	1.02	0.51–2.05
Meal/day			
	≤3 meals/day	Ref.	
	4 meals/day	0.71	0.32–1.09
	≥5 meals/day	0.84	0.37–1.32
Habit of snacking between meals			
	Yes	Ref.	
	No	2.94*	1.71–5.12
Place of meal			
	Out of home	Ref.	
	At home	1.30	0.42–4.02
Special diet in the last year			
	Weight loss	0.60	0.26–1.39
	Therapeutic	0.47	0.16–1.39
	No	Ref.	
Diet food intake			
	Daily	2.97	0.98–9.07
	Sometimes	2.68	0.91–7.91
	Never	Ref.	

* p<0.05

** Adjusted for age except for the “age group” predictive factor.

*** BMI analyzed as continuous variable.

**** *Low*: no or only primary schooling; *Medium*: secondary schooling or vocational training; *High*: university studies.

***** High-qualification occupation: managers/directors, professionals, scientists, office support staff; medium-low qualification occupation: service sector (catering, sales), craftspeople, construction, unskilled workers, homemakers.

## Discussion

This study proposes the Mediterranean Diet Serving Score, which is based on the latest MD recommendations and proved easy to apply without being less accurate than other widely accepted instruments, as demonstrated by our validation results. Other indexes based on servings have been published [28–31].

Using ROC curve methodology, it was confirmed that the MDSS can differentiate between individuals following and not following the MD pattern, using the median score as cut-off point and the MDS as reference. When the mean MDS and MDSS values were expressed in percentages to enable their comparison, the analysis (t-test for paired samples) showed that there were no significant differences between them in the mean percentage values for the total population (p = 0.341) or for each age group (adolescents: p = 0.360; adults: p = 0.273; elderly: p = 0.743) (data not shown).

The MDSS is an updated instrument that includes the most important novelties of the new MD pyramid [16]: 1) it classifies consumption frequency in servings per meal, day, or week, which is reflected in different scores; 2) the olive oil intake recommendation is changed to one serving per main meal (at the base of the pyramid with fruit, vegetables, and cereals); and 3) the recommended potato consumption frequency is reduced to ≤3 times/week (separated from the cereal group). Additionally, the proposed index considers the upper and lower recommended limits for each food group (when available), and therefore penalizes individuals both when they do not reach the recommended intake and when they exceed it. In the case of dairy products, the percentage of the study population classified as within recommendations was 52.5% with the MDS (below median dairy product consumption) but only 36.0% with the MDSS, which classified 11.5% of the population as below recommendations (2 servings/day). It should be taken into account that the MDS and other indexes of MD adherence developed before publication of the current guidelines offer less exhaustive criteria and were based on median values and the distribution in tertiles [5,6,9,12,14,32]. In the case of vegetable intake, 52.4% of the present study population was above the median vegetable intake (150 g), but this cut-off point differs from current recommendations (≥ 2 servings/meal ≈ 250–300 g/meal). Therefore, the MDSS only classified 11.4% of subjects as within recommendations (Table 3). Except in these two cases, the population was classified in a similar manner by the proposed index and the MDS, explaining the AUC value of >0.8 and supporting the validity of the new instrument.

Another novelty of the MDSS is that the total score is more influenced by meeting (or not) the recommendations of the foods at the base of the pyramid than those at its apex. Out of a maximum score of 24 points, 12 (50%) are scored when the recommended intake of fruit, vegetables, cereals, and olive oil (at the base of the pyramid) is met, 4 points (17%) for the recommended intake of dairy products and dried fruits and nuts, and 8 points (33%) for the recommended intake of legumes, potatoes, eggs, fish, white meat, red meat, sweets, and fermented drinks. Other diet quality indexes have reported a similar influence on the fulfillment of recommendations for all of these items [28,30].

The design of the MDSS permits assessment of MD adherence without the need to estimate nutrient intake. Given the importance of MUFA in cardiovascular health, the MUFA/SFA ratio is frequently considered in MD adherence patterns, with the recommendation of a high ratio because the main fat of the diet is olive oil [16]. The intake of 1 olive oil serving per meal ensures a supply of approximately 30 g MUFA/day, while the consumption of foods that are sources of SFA (dairy products, meats, and animal fat) is more limited in the MD, with a greater control over the intake of this less healthy fat. The MD recommends moderate alcohol consumption during meals (25–50 g/day for males and 5–25 g/day for females) [10], which translates into 2 servings of fermented drinks (wine or beer) a day for males and 1 for females. This study considered the consumption of fermented drinks (wine and beer) in order to reflect the importance of ethanol and non-nutritive substances (polyphenols, phytosterols, etc.), which endow the diet with antioxidant and cardioprotective properties.

Most indexes of MD adherence consider 9 items in comparison to the 14 included in the MDSS, which differentiates between the consumption of fresh fruit and dried fruit/nuts, cereals, and potatoes and red and white meats and introduces two new items to assess the intake of eggs and sweets. This permits a more accurate diagnosis of adherence to the MD by including more variables related to the characteristics of this dietary pattern [33].

In the present study, age was the main influential factor in MD adherence, as reported by other researchers [34–36]. The younger participants tended to follow a less healthy dietary pattern such as the Western diet, as reported in other studies [37–39]. A greater MD adherence was shown by individuals with lower BMI and those who did not snack between meals; this snacking, outside the five meals that should be eaten daily, is usually characterized by the intake of high-calorie foods, favoring weight gain [37]. The inverse relationship between the BMI and MD adherence may explain the protective role of this diet against chronic non-transmittable disease evidenced in numerous studies [40–42].

One study limitation was that the ROC curve was used to explore the diagnostic capacity of MDSS, considering the MDS as reference method, given the absence of a gold-standard pattern that is effective to assess MD adherence with high sensitivity and specificity values. Despite differences between the indexes, the MDSS proved able to differentiate between those following and not following the MD (using the median value as cut-off), as in the case of the MDS. In addition, the study population only included females, and the factors that determine greater adherence to the MD are likely to differ between males and females. Finally, the MDSS considers foods and food groups, whereas other diet quality indexes also include nutrients (trans-FA, SFA/MUFA ratio, ethanol). Control of the intake of these nutrients is considered in the MDSS through the limits established for certain foods, as mentioned above. This limitation could even be considered an advantage, by allowing non-specialists to apply the MDSS for MD adherence estimation in a simple and rapid manner.

The possible protective role of the MD against non-transmittable chronic diseases was not considered in the development of this index. The MDSS is proposed as an alternative to other diet quality indexes, considering the number of portions of each food group that should form part of a healthy diet in accordance with the new MD pyramid. One future approach of interest may be to explore the relationship between food groups composing the MDSS and mortality [32].

## Conclusions

The MDSS is an updated, easy, valid, and accurate instrument that assesses adherence to the MD, considering the consumption of foods and food groups per meal, day, or week. The lack of adherence of younger age groups to healthy dietary patterns, such as the MD, underlines the need to develop and apply nutritional education programs as prevention measures against the early onset of chronic non-transmittable diseases. These could have an important impact not only on the short-and long-term health of individuals but also on healthcare costs.
